# Nitrogen can improve the rapid response of photosynthesis to changing irradiance in rice (*Oryza sativa* L.) plants

**DOI:** 10.1038/srep31305

**Published:** 2016-08-10

**Authors:** Jiali Sun, Miao Ye, Shaobing Peng, Yong Li

**Affiliations:** 1 National Key Laboratory of Crop Genetic Improvement, Ministry of Agriculture Key Laboratory of Crop Ecophysiology and Farming System in the Middle Reaches of the Yangtze River, College of Plant Science and Technology, Huazhong Agricultural University, Wuhan, Hubei, China

## Abstract

To identify the effect of nitrogen (N) nutrition on the dynamic photosynthesis of rice plants, a pot experiment was conducted under two N conditions. The leaf N and chlorophyll levels, as well as steady–state photosynthesis, were significantly increased under high N. After the transition from saturating to low light levels, decreases in the induction state (*IS*%) of leaf photosynthesis (*A*) and stomatal conductance (*g*_s_) were more severe under low than under high N supply. After the transition from low to flecked irradiance, the times to 90% of maximum *A* (T_90%*A*_) were significantly longer under low than under high N supply. Under flecked irradiance, the maximum *A* under saturating light (*A*_max–fleck_) and the steady–state *A* under low light (*A*_min–fleck_) were both lower than those under uniform irradiance (*A*_sat_ and *A*_initial_). Under high N supply, *A*_max–fleck_ was 14.12% lower than *A*_sat_, while it was 22.80% lower under low N supply. The higher *IS*%, shorter T_90%*A*_, and the lower depression of *A*_max–fleck_ from *A*_sat_ under high N supply led to a less carbon loss compared with under a low N supply. Therefore, we concluded that N can improve the rapid response of photosynthesis to changing irradiance.

Photosynthesis is one of the most important biochemical processes in the world, and it is mostly studied under controlled and steady-state conditions. However, steady-state conditions are rare in nature, and growth environments, especially irradiance, are intrinsically heterogeneous in time and space within canopies^1^. Leaves within a canopy experience a highly variable light environment in magnitude (1–2000 μmol m^−2^ s^−1^) and time (seconds to minutes or longer) over the course of a day due to changes in the incoming solar irradiance, cloud cover, wind, self-shading of the upper leaves^2^,^3^. Sunflecks—relatively brief but high-intensity patches of light—have been recognised as an important energy source. It is reported that leaves, especially in the understory of the canopy, obtain 10–90% of the total photosynthetic photon flux density (PPFD) from sunflecks during 10% of the time in a day, which drives up to 65% of the total daily photosynthesis^3^,^4^,^5^.

Plants have the ability to acclimate their growth to their light environment, from whole-plant morphological changes to the differences in stoichiometry of the photosynthetic apparatus observed in leaves grown under sun and shade conditions^3^,^6^. However, this acclimation occurs over a timescale of days and weeks. In addition to this, the dynamic response of photosynthesis to rapidly changing irradiance is a quick response over a timescale of seconds to minutes, but leaf photosynthesis responds non-linearly to changes in light levels. Several minutes of low light levels will down-regulate Calvin cycle enzyme activity, and stomatal aperture; photosynthesis will thus be limited before a full induction of these photosynthesis processes occurs during a transition from low to high light levels^3^,^7^,^8^,^9^, and non-photochemical quenching (NPQ), a photoprotective mechanism to photosynthesis organs, will be improved. As a consequence, carbon loss is evident in flecked irradiance compared with uniform conditions, especially under abiotic stresses, for example high-temperature stress^5^,^10^. Moreover, photoinhibition would be occur when protective processes are either saturated or themselves damaged.

It is reported that daily carbon gain can be reduced by as much as 40% under flecked irradiance compared with uniform irradiance^10^,^11^. Similarly, plant biomass can be severely depressed under flecked irradiance^11^, especially under short-duration but high-intensity sunflecks^5^. To make full use of sunflecks, plants need rapid recovery from a low to a high photosynthetic rate when shifting to high light levels. This requires rapid activation of ribulose-1,5-bisphosphate carboxylase/oxygenase (Rubisco) and other Calvin cycle enzymes, and rapid opening of stomata^12^,^13^.

Nitrogen (N) is one of the most biologically important elements and can regulate leaf steady-state photosynthesis through several strategies, such as a large investment of leaf N to Rubisco and its involvement in stomatal opening. In order to achieve a high photosynthetic rate, approximately 75% of leaf N is allocated to chloroplasts^14^,^15^, with about 27% of this utilised in Rubisco^16^,^17^. Because of the vast investment in Rubisco and insufficient CO_2_ supply to chloroplasts, the Rubisco activation state is usually very low, especially under high N supply^18^,^19^. This suggests that, compared with a low N supply, a lower fraction of Rubisco (perhaps and other Calvin cycle enzymes) is required to be activated under high N supply for photosynthesis recovery when shifting from low to high light levels. In other words, rapid response of photosynthesis to changing irradiance may be faster under high N supply than under low N supply.

Compared with sufficient N supply, N deficiency can decrease the stomatal aperture by increasing their sensitivity to endogenous abscisic acid^20^, decreasing plant water status via depressing aquaporin expression, enhancing aerenchyma formation, and decreasing stomatal size^21^,^22^,^23^. However, it is not known whether the response of stomatal conductance to changing irradiance is related to N supply.

Moreover, in ecology, the dynamic photosynthesis of plants under flecked irradiance is generally studied using trees, which limits its relevance for the production of cereal crops, such as rice plants. In the present study, rice plants were pot-grown under both low and high N conditions, and dynamic photosynthesis was studied under simulated sunflecks. The objective was to test the hypothesis that supplying high N levels can improve the rapid response of photosynthesis to changing irradiance.

## Results

### Leaf mass per area, leaf N, and chlorophyll contents

High N supply significantly increased leaf N and chlorophyll contents (Table 1), while it significantly decreased the leaf mass per area (LMA). Compared with low N supply, leaf N contents based on leaf mass (N_mass_) and based on leaf area (N_area_) were increased by 53.8% and 42.6%, respectively, under the high N treatment. A more pronounced increase of 115.2% was observed in chlorophyll content under high N supply.

### Light and CO_2_ response curves

**Figure 1:**
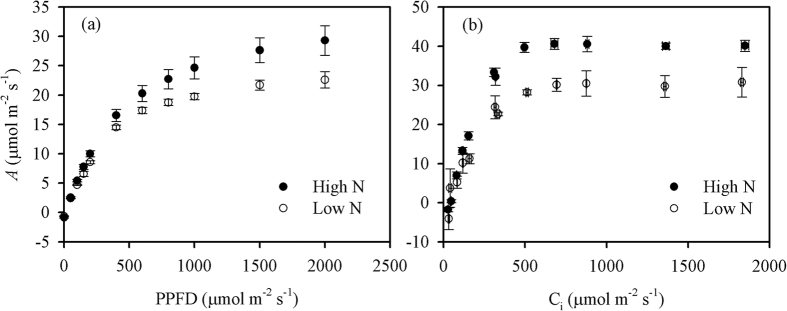
Effects of different N supplies (high N, filled cycles; low N, open cycles) on light and CO_2_ response curves. *A*, leaf photosynthetic rate; PPFD, photosynthetic photon flux density; *C*_i_, intercellular CO_2_ concentration.

Different N supplies had significant effects on both light and CO_2_ response curves (Fig. 1). Compared with 1500 μmol m^−2^ s^−1^, leaf photosynthetic rate (*A*) at 2000 μmol m^−2^ s^−1^ was not significantly improved (*P* > 0.05), which indicated that 1500 μmol m^−2^ s^−1^ was the light saturation point for photosynthesis under both N levels. With increasing CO_2_ supply, *A* increased at first and then reached a plateau when CO_2_ in the reference chamber of the photosynthesis system was between 600 and 800 μmol mol^−1^ for both N supplies. High N supply significantly increased steady-state photosynthesis under saturating light (*A*_sat_), day respiration rate (*R*_d_), light compensation point (*Q*_lcp_), apparent quantum yield (Φ), maximum carboxylation rate (*V*_cmax_), and maximum electron transport rate (*J*_max_) (Table 2). The *A*_sat_, *V*_cmax_, *J*_max_, and carboxylation efficiency (CE) increased by 47.0%, 42.9%, 48.3%, and 55.1%, respectively, which is similar to the improvement in leaf N contents. A higher increase of 110.0% was observed in *R*_d_.

### Dynamic photosynthesis

**Figure 2:**
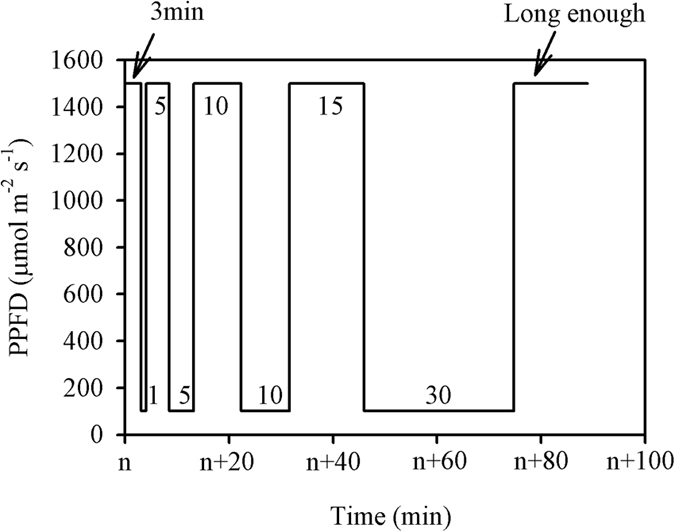
The procedure of photosynthetic induction state measurements under different durations of exposure to a low light level. Photosynthesis of the leaves was first fully induced by a prolonged light-saturated condition (n > 15, PPFD = 1500 μmol m^−2^ s^−1^), then the data were automatically recorded every 2 s. Three minutes after recording, the light was decreased immediately to 100 μmol m^−2^ s^−1^. After each low-light phase, the light was increased immediately to 1500 μmol m^−2^ s^−1^ for the leaves to be fully induced again (5, 10, and 15 min were required, respectively). There were a total of four low-light durations (1, 5, 10, and 30 min). PPFD, photosynthetic photon flux density.

**Figure 3:**
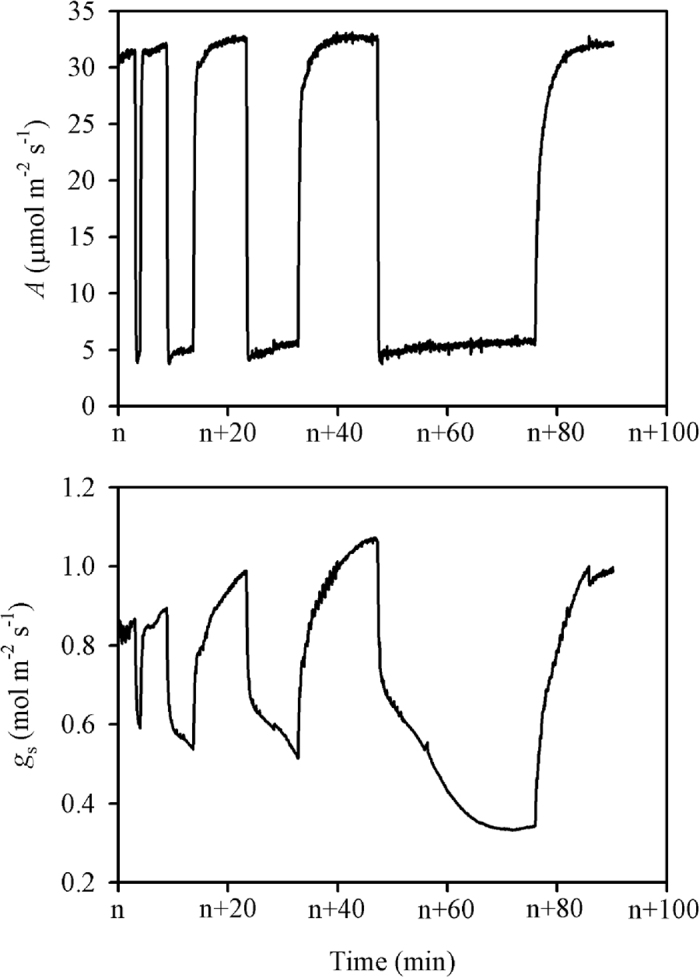
An example of the responses of the leaf photosynthetic rate (*A*) and stomatal conductance (*g*_s_) to flecked irradiance illustrated in Fig. 2.

**Figure 4:**
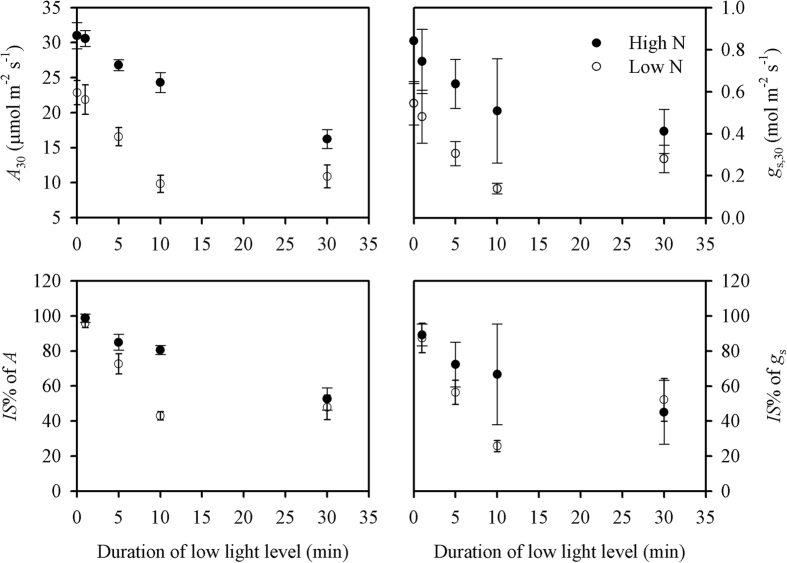
Effects of different N supplies (high N, filled cycles; low N, open cycles) on the responses of *A*_30_, *g*_s,30_, and the *IS*% values of *A* and *g*_s_ to the duration of the low light level (100 μmol m^−2^ s^−1^). *A*_30_ and *g*_s,30_ were the instantaneous photosynthetic rate and stomatal conductance, respectively, when shifting from low to high (1500 μmol m^−2^ s^−1^) light levels for 30 s. The *IS*% values of *A* and *g*_s_ were the induction state of photosynthesis and stomatal conductance after a period (1, 5, 10 and 30 min) of the low light level. The procedure for the measurement is illustrated in Fig. 2.

The response of induction state (*IS*%) to the duration of low-light conditions was measured following the procedure illustrated in Fig. 2. The data were recorded every 2 s, and an example of the responses of *A* and stomatal conductance (*g*_s_) to flecked irradiance were shown in Fig. 3. Both the instantaneous *A* and *g*_s_ at 30 s after shifting from low to high light levels (*A*_30_ and *g*_s,30_) significantly decreased with increasing periods of low light levels (Fig. 4). Interestingly, the decreases were significantly lower under high than under low N supply. The larger standard deviations of *g*_s,30_ than of *A*_30_ suggest that stomatal conductance was more variable than leaf photosynthesis.

The *IS%* of *A* and *g*_s_ both declined with the increasing period of the low light level, and the *IS%* value of *A* was always higher than that of *g*_s_, regardless of the duration of the low light level (Fig. 4). There was less of a decrease under high than under low N supply. Under low N supply, the *IS*% of both *A* and *g*_s_ reached their lowest values of 42.9% and 25.7%, respectively, after 10 min of the low light level, whereas they were 80.6% and 66.7%, respectively, under high N supply. Unexpectedly, the *IS*% values of *A* and *g*_s_ increased to 47.8% and 52.2% after 30 min of the low light level. Under high N supply, the *IS*% values of *A* and *g*_s_ gradually decreased to 52.6 and 45.0%, respectively, after 30 min of the low light level.

**Figure 5:**
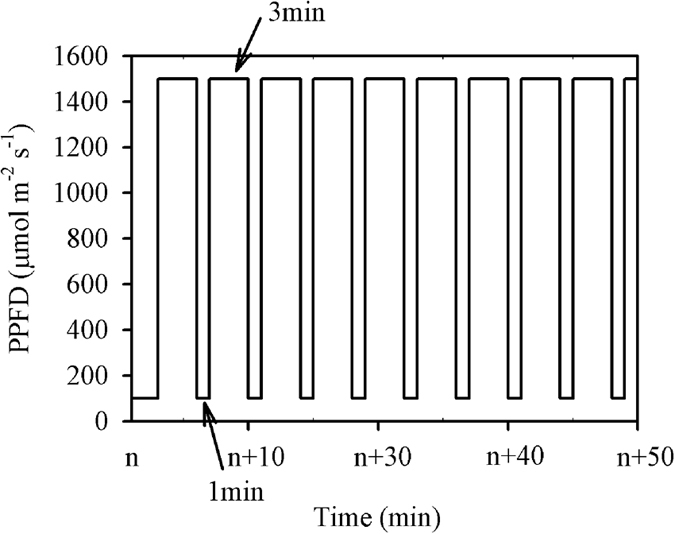
The procedure of the photosynthetic induction process measurement under periodic high light. Seedlings were kept in darkness by placing them in a controlled growth chamber (photosynthetic photon flux density (PPFD) 0 μmol m^−2^ s^−1^; temperature 28 °C; relative humidity 60%; CO_2_ concentration 400 μmol mol^−1^) from 20:00 on the previous day. Prior to the measurement, leaves were placed in the leaf chamber with a PPFD of 100 μmol m^−2^ s^−1^ for at least 15 min (n > 15), which was long enough for photosynthesis to equilibrate. Then the data were automatically recorded every 2 s, three minutes after recording, the PPFD in the chamber was set to nine 3-min flecks of 1500 μmol m^−2^ s^−1^, separated by 1-min shade periods of 100 μmol m^−2^ s^−1^.

**Figure 6:**
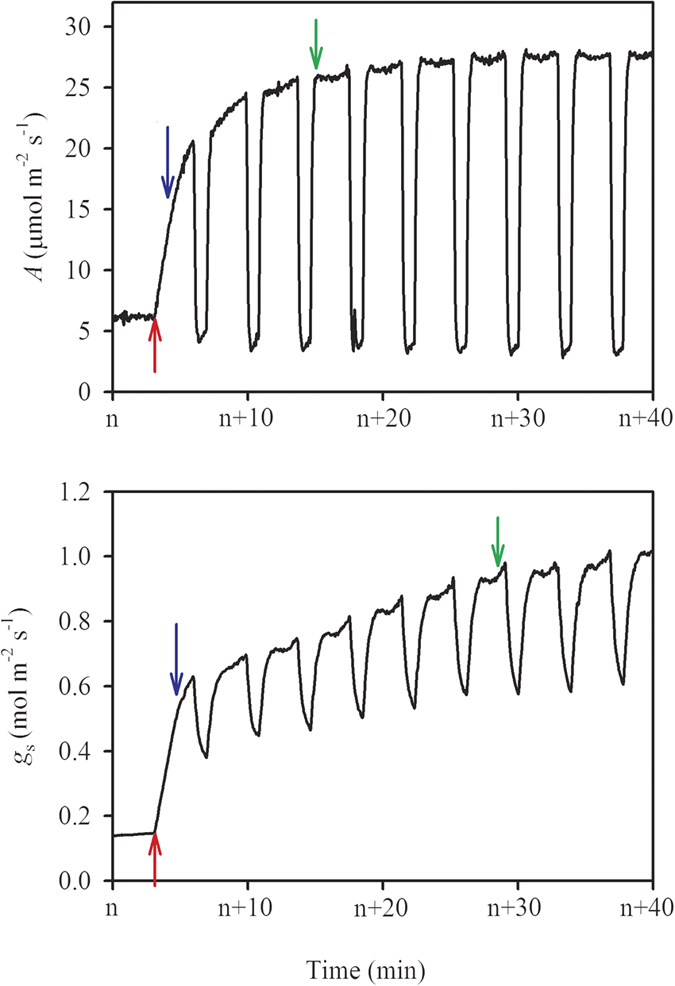
An example for the responses of the leaf photosynthetic rate (*A*) and stomatal conductance (*g*_s_) to flecked irradiance illustrated in Fig. 5. The red arrow indicate the start of first saturating light, the blue arrows indicate the data points whose instantaneous *A* and *g*_s_ reach 50% of the maximum *A* and *g*_s_, and the green arrow indicate the data points whose instantaneous *A* and *g*_s_ reach 90% of the maximum *A* and *g*_s_. Times to 50% of the maximum *A* (T_50%*A*_) and the maximum *g*_s_ (T_50%gs_) were identified as the times elapsed between the red arrows and the blue arrows, and times to 90% of the maximum *A* (T_90%*A*_) and the maximum *g*_s_ (T_90%gs_) were identified as the times elapsed between the red arrows and the green arrows.

The induction process was measured under periodic high light, following the procedure illustrated in Fig. 5. An example of the responses of *A* and *g*_s_ to the flecked irradiance is shown in Fig. 6. Prior to the measurement, leaves were placed in the leaf chamber with a PPFD of 100 μmol m^−2^ s^−1^ for at least 15 min, which was long enough for photosynthesis to equilibrate. The initial *A* and *g*_s_ values here are referred to as *A*_initial_ and *g*_s,initial_, respectively. And the maximum *A* and *g*_s_ values obtained during this process are referred to as *A*_max–fleck_ and *g*_s,max–fleck_, respectively. It was observed that high N supply significantly improved *A*_initial_, *A*_max–fleck_, *g*_s,initial_, and *g*_s,max–fleck_ (Table 3). Moreover, *A*_max–fleck_ was significantly lower than *A*_sat_, and the decrease was lower under high (14.12%) than under low (22.80%) N supply (Tables 2 and 3). Similarly, the steady-state *A* at low light periods of the flecks (*A*_min–fleck_) was significantly lower than *A*_initial_, but the decreased percentage was similar between the two N levels (high N, 31.89%; low N, 30.66%).

Times to 50% and 90% of the maximum photosynthetic rate (*T*_50%*A*_ and *T*_90%*A*_, respectively) were identified as the period between the start of the first high light level and the time when the first data point exceeded each of the values in turn (illustrated in Fig. 6). Times to 50% and 90% of the maximum stomatal conductance (*T*_50%*gs*_ and *T*_90%*gs*_) were calculated similarly. It showed that high N supply significantly decreased *T*_90%A_, but did not affect *T*_50%A_, *T*_50%gs_, or *T*_90%gs_ (Table 3). The time required for photosynthesis recovery was lower than that required for stomatal conductance recovery (*T*_50%A_ *versus T*_50%gs_, *T*_90%A_ *versus T*_90%gs_), but only significant under high N supply. Post-irradiance CO_2_ fixation and CO_2_ burst consisted of a small amount of integrated carbon gain, averaging 5.76% and 0.36%, respectively, under the two N supplies.

### Integrated carbon gain and carbon loss

Integrated carbon gain was calculated as the integrated photosynthesis within 36 min from shifting to a high light level to the end of the ninth low light period (Figs 5 and 6). Carbon loss is usually calculated as the induction loss relative to *A*_max–fleck_; in the present study, *A*_max–fleck_ and *A*_min–fleck_ were both observed to be lower than their corresponding steady-state photosynthesis *A*_sat_ and *A*_initial_ values (Tables 2 and 3). If photosynthesis was fully induced during this photosynthetic induction procedure, the potential carbon gain should be calculated using *A*_sat_ and *A*_initial_ rather than *A*_max–fleck_ and *A*_min–fleck_ to avoid underestimation, 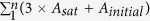, where n is the number of flecks in Fig. 5 which is 9 in the present study. Carbon loss is the difference between the potential and integrated carbon gains.

As expected, the high N supply significantly increased both the integrated and the potential carbon gains (Table 4). The potential carbon gain was substantially higher than the integrated carbon gain under both N supplies, which resulted in a significant carbon loss. Carbon loss was more severe under low than under high N supplies.

## Discussion

The difference in *T*_90%A_ supported our hypothesis that high N supply can improve the rapid response of photosynthesis to changing irradiance. Because of the rapid induction process under high N supply from the low light level, its carbon loss was significantly lower than under the low N supply. The *A*_max–fleck_ and *A*_min–fleck_ were observed to be significantly lower than their corresponding steady-state photosynthesis values (*A*_sat_ and *A*_initial_). Moreover, the depression of *A*_max–fleck_ relative to *A*_sat_ was lower under high (14.12%) than under low (22.80%) N supply. To the best of our knowledge, this is the first study to systemically investigate the effects of different N supplies on dynamic photosynthesis.

### Steady-state photosynthesis

The LMA is an important leaf anatomical trait, which can be calculated by dividing the leaf dry mass by the leaf area. The response of the LMA to N supply has been intensively studied previously^24^,^25^,^26^, which showed that the LMA can be either increased or decreased with an increased N supply, or be independent of the N supply. In the present study, the LMA was decreased under high N supply, which resulted in a relatively smaller increase in percentage of N_area_ in comparison with N_mass_ (Table 1). Because of the large investments of N in electron transport proteins (about 7% of leaf N) and Rubisco, the primary enzyme of the Calvin cycle, leaf photosynthesis is usually positively related to leaf N content. In the present study, *A*_sat_, *V*_cmax_, and *J*_max_ increased proportionally to N_area_ under high N supply (Tables 1 and 2).

### Dynamic photosynthesis

#### Photosynthesis deactivation and induction process

The photosynthetic *IS*% decreased with the increasing periods of low light of between 1 and 30 min. Possessing a higher *g*_s_, the rice plants in the present study had a higher *IS*% relative to rainforest trees^11^,^27^, probably because of the positive correlations between *IS*% and *g*_s_^28^. Changes in *IS*% are reported to be related to plant species and growth environments, namely CO_2_ concentration, soil moisture, temperature, and irradiance^5^,^11^,^27^,^28^. In the present study, the reduction of *IS*% was demonstrated to be related to N supply (Fig. 4). The more severe decrease of *IS*% under low N supply was caused, at least in part, by more rapid stomatal closure.

After a transition from low to high light levels, photosynthetic induction proceeds, involving several induction processes (e.g. activation of Calvin cycle enzymes and rapid opening of stomata) operating at different timescales. Lawson *et al*.^3^ stated that 1 to 10 min is required to activate Calvin cycle enzymes via the thioredoxin system, except for Rubisco, which requires up to 30 min for full activation; stomatal opening is even slower than the activation of Rubisco. Allen and Pearcy^28^ showed that the limitation of biochemical enzymes during photosynthesis induction was significantly more severe than that of stomatal conductance, although the activation of these two processes seemed to be highly coordinated. In the present study, the time required for photosynthesis induction (*T*_50%A_ and *T*_90%A_) was lower than that required for stomatal conductance (*T*_50%gs_ and *T*_90%gs_), but only significant under high N supply (Table 3), which suggests that the biochemical induction rate was faster than the stomatal conductance rate under high N supply but not under low N supply.

Why was *T*_90%A_ lower under high than under low N supply? Due to the insufficient CO_2_ concentration in chloroplasts and the low CO_2_ affinity of Rubisco, Rubisco may operate significantly below its potential catalytic capacity in C_3_ plants such as rice. The Rubisco activation state usually decreases with increasing N supplies^19^,^29^,^30^, which suggests that, compared with plants grown under low N conditions, a lower fraction of Rubisco needs be activated to achieve photosynthesis under high N supply. This might be one of the reasons why *T*_90%A_, which matches the timescale required for Rubisco activation, was lower under high than under low N conditions. Further detailed research is needed to investigate the effects of N supplies on the photosynthesis induction rate.

#### Comparison with steady-state photosynthesis

When receiving a sudden increase in irradiance from sunflecks, shade-adapted leaves require appreciable periods of time to active the photosynthesis apparatus, and NPQ is very important to protect photosynthesis apparatus from damage during this period^31^. It showed that photosystems I and II could not be sufficiently activated under simulated sunflecks especially with a short duration of saturating irradiance^31^, probably because of the deleterious effect of photoprotection to CO_2_ assimilation^32^. The duration of saturating irradiance in the study of Sejima *et al*.^31^ was relatively short however, from 10 to 300 ms. Compared with the present study, a similar fleck pattern (3 min flecks of high light, separated by 1 min of low light) was used in the study of Leakey *et al*.^11^, which showed that the maximum photosynthesis during flecks (4.91 μmol m^−2^ s^−1^) was lower than the steady-state photosynthesis (6.08 μmol m^−2^ s^−1^) calculated from light response curves (see Tables 3 and 5 in Leakey *et al*.^11^). Similarly, *A*_max–fleck_ and *A*_min–fleck_ were significantly lower than *A*_sat_ and *A*_initial_, respectively, in the present study. This suggests that the photosynthesis apparatus could not be sufficiently activated under flecked irradiance, which would inevitably induce carbon loss compared with uniform irradiance.

#### Carbon gain and loss

The lag of photosynthesis after the rise in PPFD and the post-irradiance CO_2_ burst are two carbon-loss processes in addition to respiration, and post-irradiance CO_2_ fixation is a compensating strategy for carbon gain. In the present study, the post-irradiance CO_2_ fixation and CO_2_ burst were not significantly different between the two N supplies, and they comprised a small fraction (5.76% and 0.36%, respectively) of the integrated carbon gain (Table 3). Therefore, the lag of photosynthesis after the transition from low to high light levels, as well as the differences between dynamic and steady-state photosynthesis levels (*A*_min–fleck_ *versus A*_initial_, *A*_max–fleck_ *versus A*_sat_), were the two major reasons for the discrepancy between the integrated and the potential carbon gains. The lower carbon loss under high N supply was mostly caused by the relatively lower discrepancy between *A*_max–fleck_ and *A*_sat_ (Tables 2 and 3), the lower photosynthetic induction loss rate under shade (Fig. 4), and rapid photosynthesis recovery rate (*T*_90%A_) after the transition to the high light level (Table 3). It should be noted that a small increase in photosynthesis can result in a greater difference in plant biomass^3^.

### Implication in the field

Dynamic photosynthesis is mostly investigated in forest trees; studies on cereal crops are rare, despite the fact that the leaves of rice plants in the field also experience a highly variable light environment over the period of a day. Studies on the environmental determination of dynamic photosynthesis can provide novel information to improve natural photosynthesis and crop yield. The present study has shown that N can improve both steady-state and dynamic photosynthesis in rice. Further research should be conducted to investigate the spatial and temporal variations of irradiance in crop canopies, and this knowledge could be coordinated with the vertical distribution of leaf N to improve both light and N use efficiency.

## Materials and Methods

### Plant materials and N treatments

After germination on a moist filter on 11 June 2015, seeds of the inbred rice cultivar *Oryza sativa* L., ssp. indica, *cv*. ‘Huanghuazhan’ (HHZ), which is widely grown in Hubei province, were transferred to nursery plates. When the seedlings had developed an average of 2.5 leaves, they were transplanted to 11.0 L pots with a density of three hills per pot and two seedlings per hill. Each pot was filled with 10.0 kg of soil, and phosphorus (P) and potassium (K) were each applied at a rate of 1.5 g pot^−1^. N was applied at the rates of 0.12 and 0.80 g N pot^−1^ under low N and high N treatments, respectively. Fertilisers were applied by mixing them into the soil. Plants were watered daily and a minimum layer of 2 cm water was maintained to avoid drought stress. Pests were intensively controlled using chemical pesticides. The soil used in this study has the following properties: pH 7.1, 6.7 g kg^−1^ organic matter, 6.27 mg kg^−1^ Olsen-P, 129 mg kg^−1^ exchangeable K, 0.63‰ total N.

The experiment was conducted outdoors in Huazhong Agricultural University (114.37°E, 30.48°N) in Wuhan city, Hubei province, China. Measurements were conducted at the tillering stage, from 35 d after germination.

### Gas exchange measurements

#### Steady-state gas exchange measurement

One day before the gas exchange measurement started, rice seedlings were removed to a controlled growth chamber (PPFD 1000 μmol m^−2^ s^−1^ at the leaf level; temperature 28 °C; relative humidity 60%; CO_2_ concentration 400 μmol mol^−1^). Gas exchange measurements were conducted on the newest fully expanded leaves using a portable photosynthesis system (LI-6400XT; LI-COR Inc., Lincoln, NE, USA) between 09:00 h and 16:00 h. Prior to the measurements for light response curves, leaves were placed in the leaf chamber for at least 15 min at a PPFD of 1500 μmol m^−2^ s^−1^, a CO_2_ concentration in the reference chamber of 400 μmol mol^−1^ with a CO_2_ mixture, a leaf temperature of 28 °C, and a leaf-to-air vapour pressure deficit of 1 kPa. After equilibration to a steady state, data were recorded and the measured *A* was used to define the steady-state photosynthesis (*A*_sat_). Thereafter, the PPFD was controlled across a series of 2000, 1500, 1000, 800, 600, 400, 200, 150, 100, 50, and 0 μmol m^−2^ s^−1^ to measure the light-response curves. The Φ was calculated by linear regression of the data points on the light-limited part (from 200 to 0 μmol m^−2^ s^−1^) of the light-response curves. The *Q*_lcp_ and *R*_d_ were calculated by fitting a non-rectangular hyperbola.

Prior to the measurements of the CO_2_-response curves, leaves were placed in the leaf chamber for at least 15 min under the above-mentioned conditions. After equilibration to a steady state, CO_2_ concentration in the reference chamber was controlled across a series of 400, 200, 150, 100, 50, 25, 400, 600, 800, 1000, 1500, and 2000 μmol mol^−1^ with a CO_2_ mixture to measure the CO_2_-response curves. Carboxylation efficiency was calculated by linear regression of the data points when the CO_2_ concentration in the reference chamber was ≤200 μmol mol^−1^. The *V*_cmax_ and *J*_max_ were calculated according to the FvCB model and its modification^33^,^34^.

#### Dynamic gas exchange measurement

Before the measurement of dynamic gas exchange, the response time of the gas exchange apparatus was checked. A quick response time of 5 s at a flow rate of 500 mL min^–1^ was observed, which was similar to that in other studies^5^,^11^. Estimation of *IS*% and the post-irradiance CO_2_ fixation and CO_2_ burst were calculated after raw output was corrected for the system lag time.

Leaf instantaneous photosynthesis capacity will be down-regulated under dark or low light levels, and a longer duration of dark or low-light conditions will result in a more severe deactivation of photosynthesis. The response of *IS*% to the duration of low-light conditions was measured under periodic high light according to the procedure described by Timm *et al*.^2^. The leaf photosynthetic rate at the end of the first saturated light period was similar to *A*_sat_ calculated from light-response curves, because they were both fully induced at 1500 μmol m^−2^ s^−1^ for at least 15 min (Figs 2 and 3). Hereafter, the *A* at the end of the first saturated light period is also referred to as *A*_sat_. Similarly, *g*_s,sat_ is the *g*_s_ at the end of the first saturated light period. When shifting from low to high light levels, *A* at first increased and then reached a plateau. The *IS*% value of *A* was calculated as


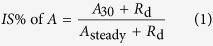


*g*_s_ at this time was referred to as *g*_s,30_. *g*_s_ at darkness is nearly 0 mol m^−2^ s^−1^, so the *IS*% value of *g*_s_ can be calculated as


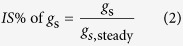


The photosynthetic induction process has a lag period after the rise in PPFD, and photosynthetic CO_2_ fixation continues for several seconds when shifting from high to low light levels^11^,^35^. The induction process was measured according to the procedure described by Allen and Pearcy^28^ and Leakey *et al*.^11^. Briefly, seedlings were kept in darkness by placing them in a controlled growth chamber (PPFD 0 μmol m^−2^ s^−1^; temperature 28 °C; relative humidity 60%; CO_2_ concentration 400 μmol mol^−1^) from 20:00 on the previous day until the measurement was started at 09:00. After a prolonged low light period (>15 min), the PPFD in the chamber was set to nine 3 min flecks of 1500 μmol m^−2^ s^−1^, separated by 1 min shade periods of 100 μmol m^−2^ s^−1^. It can be observed from Fig. 6 that, from the sixth fleck, the instantaneous *A* responsed rapidly to irradiance and can almost reach the *A*_max–fleck_, although *g*_s_ at each end of the flecks increased gradually during the measurement. When shifting from high to low light levels, *A* dropped below *A*_initial_ because of the post-irradiance CO_2_ burst, and finally returned to a steady-state rate, *A*_min-fleck_. The *A*_min-fleck_ does not necessarily equal *A*_initial_ (see Fig. 5 in Leakey *et al*.^11^). The post-irradiance CO_2_ fixation and CO_2_ burst were calculated according to Leakey *et al*.^11^.

### Measurements of leaf N and chlorophyll contents

Leaves were detached immediately after gas exchange measurements, followed by leaf area measurement using a LI-Cor 3000C (LI-CORInc., Lincoln, NE, USA). They were then oven-dried to achieve a constant weight at 80 °C; after which the leaf dry mass was recorded. The leaf mass per area was calculated as the ratio of the leaf dry mass to its corresponding leaf area. Leaf N content based on leaf mass was measured with a stable isotope ratio mass spectrometer (IsoPrime100 IRMS, Isoprime Ltd, UK), and N_area_ was calculated by multiplying N_mass_ with LMA. The chlorophyll content of newly expanded leaves was extracted using 80% acetone and quantified using the colourimetry method (Arnon, 1949).

### Statistical analysis

One-way analysis of variance (ANOVA) and the least-significant difference (LSD) test were used to assess each of the parameters using Statistix 9 software (Analytical Software, Tallahassee, Florida, USA).

## Additional Information

**How to cite this article**: Sun, J. *et al*. Nitrogen can improve the rapid response of photosynthesis to changing irradiance in rice (*Oryza sativa* L.) plants. *Sci. Rep.* **6**, 31305; doi: 10.1038/srep31305 (2016).

## Figures and Tables

**Table 1:** Effects of N supplies on leaf mass per area (LMA), mass-based and area-based leaf N contents (N_mass_ and N_area_), and chlorophyll content.

Treatments	LMA (g m^−2^)	N_mass_ (%)	N_area_ (g m^−2^)	Chlorophyll content (mg g^−1^)
High N	41.9 ± 1.8 b	3.50 ± 0.02 a	1.47 ± 0.07 a	1.01 ± 0.21 a
Low N	45.2 ± 0.9 a	2.28 ± 0.02 b	1.03 ± 0.02 b	0.47 ± 0.04 b

Note: Data are presented as means ± SD with three replications, and each replication contains at least three newly expanded leaves; data followed by different letters are significant at the *P* < 0.05 level.

**Table 2:** Effects of different N supplies on the steady-state photosynthetic rate under saturating irradiance (*A*_sat_), day respiration (*R*_d_), light compensation point (*Q*_lcp_), apparent quantum yield (Φ), maximum Rubisco carboxylation capacity (*V*_cmax_), maximum electron transport capacity (*J*_max_), and carboxylation efficiency (CE).

Treatments	*A*_sat_ (μmol m^−2^ s^−1^)	*R*_d_ (μmol m^−2^ s^−1^)	*Q*_lcp_ (μmol m^−2^ s^−1^)	Φ	*V*_cmax_ (μmol m^−2^ s^−1^)	*J*_max_ (μmol m^−2^ s^−1^)	CE
High N	33.35 ± 1.06 a	0.422 ± 0.075 a	7.81 ± 0.99 a	0.054 ± 0.003 a	110 ± 4 a	218 ± 6 a	0.159 ± 0.018 a
Low N	22.68 ± 0.45 b	0.201 ± 0.089 b	4.42 ± 1.92 b	0.045 ± 0.002 b	77 ± 6 b	147 ± 12 b	0.102 ± 0.004 b

Note: Data are presented as means ± SD with three replications; data followed by different letters are significant at the *P* < 0.05 level.

**Table 3:** The effects of different N supplies on steady-state photosynthesis under a low light level (*A*_initial_), maximum photosynthetic rate under flecks (*A*_max–fleck_), minimum photosynthetic rate under flecks (*A*_min–fleck_), steady-state stomatal conductance under a low light level (*g*_s,initial_), maximum stomatal conductance under flecks (*g*_s,max–fleck_), times to 50% and 90% of *A*_max–fleck_ (*T*_50%A_ and *T*_90%A_), times to 50% and 90% of *g*_s,max–fleck_ (*T*_50%gs_ and *T*_90%gs_), post-irradiance CO_2_ fixation, and CO_2_ burst.

Parameters	High N	Low N
*A*_initial_ (μmol m^−2^ s^−1^)	5.99 ± 0.15 a	4.99 ± 0.88 b
*A*_max–fleck_ (μmol m^−2^ s^−1^)	28.64 ± 1.45 a	17.51 ± 404 b
*A*_min–fleck_ (μmol m^−2^ s^−1^)	4.08 ± 0.30 a	3.46 ± 0.89 a
*g*_s,initial_ (mol m^−2^ s^−1^)	0.16 ± 0.02 a	0.09 ± 0.04 b
*g*_s,max_ (mol m^−2^ s^−1^)	1.28 ± 0.20 a	0.50 ± 0.16 b
*T*_50%A_ (min)	1.67 ± 0.21 a	1.47 ± 0.44 a
*T*_90%A_ (min)	10.88 ± 2.02 b	17.91 ± 2.79 a
*T*_50%gs_ (min)	2.56 ± 0.41 a	2.20 ± 0.66 a
*T*_90%gs_ (min)	22.48 ± 15.51 a	19.56 ± 3.69 a
Post-irradiance CO_2_ fixation (%)	5.51 ± 0.20 a	6.01 ± 1.55 a
Post-irradiance CO_2_ burst (%)	0.17 ± 0.05 a	0.54 ± 0.45 a

Note: Data are presented as means ± SD with three replications; data followed by different letters are significant at the *P* < 0.05 level.

**Table 4:** The effects of different N supplies on integrated carbon gain, potential carbon gain, and carbon loss.

Treatments	Integrated carbon gain (μmol m^−2^)	Potential carbon gain (μmol m^−2^)	Carbon loss (%)
High N	42 498 ± 1666 a	57 104 ± 101 a	25.58 ± 2.82 b
Low N	24 938 ± 6507 b	42 574 ± 367 b	41.46 ± 15.03 a

Note: Data are presented as means ± SD with three replications; data followed by different letters are significant at the *P* < 0.05 level.
